# Reframing Social and Emotional Development of the Gifted

**DOI:** 10.3390/bs14090752

**Published:** 2024-08-27

**Authors:** Robert J. Sternberg

**Affiliations:** Department of Psychology, College of Human Ecology, Cornell University, Ithaca, NY 14853, USA; rjs487@cornell.edu

**Keywords:** giftedness, socioemotional development, general mental ability, social intelligence, emotional intelligence, practical intelligence

## Abstract

This essay questions the framing of socioemotional development as a separate concomitant of cognitive development in gifted individuals. Rather, it argues, first, that socioemotional development of the gifted is not separate from giftedness. Second, socioemotional development is not even cleanly and clearly separable from cognitive development. Third, giftedness and even intelligence do not reside inside the person—they are not personal properties but rather interactions of persons with tasks and situations. In sum, giftedness needs to be viewed in a holistic context that encompasses integrated cognitive, socio-emotional, task, and situational contextual elements.

## 1. Introduction

In the times of Lewis Terman, an early founder of the gifted-child movement in the United States, it was a major achievement to show that gifted children were not, as often had been assumed, social misfits or even freaks but were, for the most part, youngsters who were socially well-developing members of society [1]. This mistaken assumption was unfortunate [2]. Much of the research on social and emotional development of gifted and talented individuals since then has shown that, although gifted children have challenges—for example, envy from others, challenges in fitting into groups who do not share their intellectual interests, feelings of threat aroused in some teachers—the children do quite well socially, for the most part (e.g., [3,4,5]).

Most of this research, however, makes a different assumption that is challenged in this essay. The assumption is that gifted children should be identified primarily on the basis of cognitive skills, and the question to be asked, then, is how things are going with their concurrent but largely identification-irrelevant social and emotional development. Are they doing all right socially and emotionally, despite being gifted, or perhaps because of it? Indeed, most measures used in identification, such as intelligence tests, achievement tests, and school grades, are either explicitly cognitive or, when there are social factors intruding, as in grade point averages (GPAs), these factors are viewed largely as “contaminating” factors that take away from the “purity” of the measures.

This essay questions three assumptions in much of the literature:

1. Socioemotional development of the gifted is separate from giftedness;
2. Socioemotional development is even cleanly separable from cognitive development;
3. Giftedness and even intelligence somehow reside inside the person—they are personal properties.

These are three common assumptions that are questionable and, I argue, incorrect in this framing of giftedness and its development. Consider each assumption in turn.

## 2. The Assumption That Socioemotional Development of the Gifted Is Separate from Giftedness

The first assumption is that social and emotional development of the gifted is separate from whatever it is that makes them gifted—that it is something one wants to study as a discrete variable separate from giftedness. Yet, in some modern and even not so modern theories of intelligence, social, emotional, interpersonal, intrapersonal, practical, and other kinds of intelligence have been identified and construct-validated in ways that suggest that social and emotional development is not some kind of “sidekick” of gifted development, but rather may be a basis for gifted development (e.g., [6,7,8]). In adults, certainly, cognitive intelligence in the absence of reasonable levels of social, emotional, and practical intelligence can be a disaster in the making [9].

Terman [10] followed Binet and Simon [11,12] in his belief that the tests that matter for predicting school achievement, and also later-life accomplishment, should be cognitive. This idea fit in with Spearman’s [13,14] idea that there is a general factor of intelligence that permeates all behavior that is needed for cognitive achievement. Spearman [15] even proposed a model of how general intelligence operates, which also was an information-processing model. This notion of information processing underlying intelligence has been applied worldwide (see [16,17]), but it is limited.

Although Spearman also proposed “mental energy” as a basis for general intelligence [14], this concept has always seemed rather vague and ill-defined and has not generated much research, unlike the 1923 information-processing model, because it has remained unclear, over a century, what mental energy might be or how it would be measured.

There are a number of kinds of intelligence that should not be viewed as collateral to giftedness but rather as ways in which giftedness can and should be identified. These kinds of intelligence are socioemotional as well as cognitive, but oddly, the fact that they are partially socioemotional seems, historically, to have excluded them from being considered as part of intelligence and of what should serve as a basis for identifying the gifted. Educators have taken seriously Boring’s [18,19] stipulation that intelligence is what IQ tests measure, and so, good-bye to anything that is not that.

Wechsler [20] took the view that “Social intelligence is just general intelligence applied to social situations” (p. 75). This view is historically important because it became what appears to be the predominant view [21,22,23,24,25,26,27]. Even James Flynn, famous for having observed that IQs rose 30 points in the 20th century, and who had a very broad view of what intelligence is, accepted IQ as the basic measurement of the construct [28,29,30].

On the one hand, it is understandable why general intelligence, or *g*, would receive the lion’s share of attention, at least among psychometricians. Measures of general intelligence are quite reliable over time—at least as much as any identifiable psychological trait—and the measures are predictively valid for many purposes [26]. They usually yield a strong and identifiable single factor—the first one in an unrotated exploratory factor analysis. The measures are relatively low in bias—so long as one does not take into account bias in the criteria against which they are validated [31,32].

The criterion bias is a serious one, and one that gives cognitive measures an unfair advantage when they are being considered as the “go to” measures for identifying and assessing people as gifted. In the United States, for example, educational institutions at all levels use standardized tests that, whatever they are called, essentially measure general mental ability (sometimes referred to as “GMA”, or *g*) plus other subsidiary knowledge and skills. Such measures have been validated over the years by showing that performance on them predicts future success of diverse kinds, the basis for much of the argument for the importance of general mental ability in *The Bell Curve* [24].

The problem is that performance on the criterion measures used to validate cognitive tests is affected, perhaps substantially, by performance on the predictor measures [31]. This is a clear statistical confounding, and yet it goes ignored or unnoticed in much of the validation literature. Consider, as an example, a young person who is admitted to a prestigious college despite a middling record in school. Some schools, such as one at which the author of this article was an administrator, are prepared to admit college students *solely* on the basis of a standardized test score. And, of course, high-IQ societies, of which there are many, are proud to admit individuals only on the basis of IQ. That is their *raison d’être,* so to speak, and of course, that is their prerogative. The membership then can be used to embellish resumes, if the individual indeed believes that such a membership will embellish their resume.

Many of the most prestigious companies, at least in the United States, only recruit college graduates from highly prestigious universities where standardized test scores and other measures of GMA often form a major basis for admission. Put concretely, it may be easier to obtain a prestigious, high-paying placement out of schools with high-GMA students, such as Harvard or Yale, than out of open-admission schools that take anyone who applies so long as they have a high-school degree. These schools may not even require the high-school degree. This is not to say that high-GMA individuals will not perform well in their subsequent jobs or in their life. But part of their higher level of performance, starting in elementary school, may be a result of having been given opportunities that other students are not given. After a while, it becomes impossible to separate the effect of high GMA from the effect of the privileges a society bestows on a high-GMA student.

All societies, of course, have given advantages to certain individuals over others [32]. The advantage may be based on GMA, or socially defined race, or religious preference, or societally recognized “royal birth”, or socioeconomic status, or whatever. But advantages of one kind tend to open up advantages of other kinds in a nonlinear but rather comprehensive and integrative way. GMA itself seems to reflect socioeconomic advantage. The squared correlation between economic advantage, as measured by family income and SAT test scores (a measure of GMA), is in the order of 0.95 [33]. This correlation leaves totally unclear the direction(s) of causality. High IQ may lead to better family income; better family income may lead to higher IQ; both may be dependent on something else; or most likely, all these possibilities are true and interactive, to some extent.

So, if one asks how socially and emotionally based aspects of intelligence and of achievement came to play, at best, second fiddle, especially in the identification and development of gifted children, one will have to understand the history. Moreover, there is an ugly aspect of this history that has been pointed out by many [34,35,36,37]. IQ can be a proxy for many things and has been used in part to perpetuate socially defined racial, ethnic, national, and other bases of discrimination. The societies most culpable for such use of IQ have been ones that emphasize cognitive rather than socioemotional skills in their conceptions of competence and even intelligence [38].

For this advantaging of cognitive over socioemotional measures in the identification of giftedness to continue requires a rather narrow conception of cognitive skills. Gardner [6] and Sternberg [7,8] have offered broader conceptions of intelligence, as did Guilford [39,40], from a psychometric point of view. But these broader conceptions have given way to contemporary models that emphasize narrower cognitive skills [41,42,43].

Socioemotional skills are harder to measure validly and reliably than cognitive skills and likely are more variable. Although they are valued quite a bit in adult life, they are more notable in school by the kind of the kind of extreme deficit that leads to trouble, or even probation or expulsion, than they are by leading to better recorded performance. They thus tend to count less in the academic record of the gifted, even though later in life they will matter so much. What kinds of socioemotional skills are referred to here? Consider specifically other kinds of intelligence besides the cognitive that draw on socioemotional skills [44].

### 2.1. Social Intelligence

The idea of a *social intelligence* is actually a very old idea, going back to the early days of psychological theorizing about intelligence. The idea is still used in modern times [45]. The term, “social intelligence”, was introduced by Dewey [46] and was taken up by Lull [47]. The definition that has resonated over time is that of Edward Thorndike [48]: “By social intelligence is meant the ability to understand and manage men and women, boys and girls—to act wisely in human relations” (p. 228). Robert Thorndike, Edward Thorndike’s son, was also among the first to seek to measure social intelligence [49], although measurement of social intelligence never took off in the field of psychology the way that measurement of cognitive intelligence did. There have been other definitions as well (see [45], such as that of Moss and Hunt [50] as the “ability to get along with others” (p. 108). Vernon [51] defined social intelligence as the “ability to get along with people in general, social technique or ease in society, knowledge of social matters, susceptibility to stimuli from other members of a group, as well as insight into the temporary moods or underlying personality traits of strangers” (p. 44). But the view that predominated was that of Wechsler [20]—that social intelligence is just cognitive intelligence used socially.

Guilford’s [39,40,52] model of the Structure-of-Intellect had a behavioral content dimension that could be used to measure social intelligence. Guilford was among the first, then, fully to integrate social intelligence into an overall model of intelligence. Guilford even devised measures of the behavioral facet.

Why did the behavioral facet proposed by Guilford not resonate in psychology or beyond? There are at least three possible reasons.

One reason almost certainly is that Guilford’s model, although popular during the early decades of the latter half of the twentieth century, lost much of its appeal as a result of the research of John Horn [53,54], which suggested that much of the empirical factor-analytic support for Guilford’s model was artifactual. In particular, the form of factorial rotation Guilford used, called “Procrustean rotation”, so capitalized on chance that it could support the model even with random data or random theories!

A second reason is that the Guilford model, eventually ending up with 180 supposedly orthogonal psychometric factors, was exceedingly complex and did not lend itself well to measurement. Who could realistically administer 180 distinct psychometric subtests to their participants? Although parsimony is perhaps not necessary for all psychological theories, seemingly endless complexity is not always appreciated.

A third reason is probably that there was, by the time Guilford proposed his model, a bias toward keeping intelligence as a cognitive trait. A large volume of literature had evolved characterizing intelligence as cognitive [55,56], and it probably would have taken a lot of work to convince a scholarly audience that the concept should be extended. That was true in the latter half of the twentieth century, and it is still true today.

Guilford [39] was not the first to develop tests of social intelligence. Probably the first well-known attempt was the George Washington Social Intelligence Test [50,57,58]. The test used social content, but such content can be challenging because of its being associated with culture and secular time. In my own lab, we attempted to use the test in some of our research and found many of the items to be outdated and even quaint. When one thinks of social customs today and a century ago, they just are not so similar. One of the tests was sense of humor, and what is considered funny changes over time and place, and even can be unevenly distributed within a given time and place, as my own family’s reactions to my jokes have shown. This test seemed promising at first, showing correlations with external criteria such as college extracurricular performance and occupational performance. But the test was also fairly robustly correlated with general intelligence [49], which was viewed as problematic.

A challenge that has faced socioemotional measures, whether used for identification of giftedness or for anything else, is that the measures tend to show significant and often moderate (or higher) correlations with cognitive tests [45]. Because the cognitive tests have historical precedence, and because they correlate with so many things, any new theory or test based on that theory will need to show a kind of incremental validity—how much does the new theory or test account for that is not already accounted for by present theories or tests? In the case of measures for identification of the gifted, the question, then, is whether measures of socioemotional skills add significantly—in the sense of both statistical and practical significance—to the identification procedure that they are worth the cost and effort of being added to a battery for identification of the gifted.

The socioemotional theories and tests face barriers beyond those of historical precedent and the robust correlations of cognitive tests with so many things. For one thing, it is harder to measure socioemotional skills in a highly reliable way than it is to measure cognitive skills [59]. Although both kinds of skills change over time, including with respect to rank orders of people taking the tests, socioemotional skills probably change more because they are psychologically more labile and more situation-specific. One probably shows different sets of socioemotional skills with one’s teachers versus one’s classmates, but the cognitive skills are likely to be more nearly common across the situations.

At some level, both cognitive and socioemotional characteristics can be labile. All abilities are, to some extent, interactions between persons x tasks x situations [60]. Abilities do not merely rest within the person: Someone might be strong in their performance at verbal tasks but not spatial tasks, or vice versa; similarly, someone might be strong in their performance if they are relaxed but not if they are anxious, or vice versa. But socioemotional characteristics can vary greatly even across two different persons with whom one has very different relations. So, finding an average level can be rather challenging.

One then might ask, of course, if socioemotional characteristics are labile, why bother to measure them at all? The reason was recognized even by Terman in 1925 and was pointed out more recently by Brooks [61], based on findings on gifted children as adults, and echoing findings reported throughout the literature [62,63]. As an adult, cognitive skills carry one only so far [64,65]. If one cannot work in teams, collaborate on projects, maintain good social relations with supervisors and coworkers, act respectfully and with grace toward others, it is very hard to succeed in a job, or even, often, to keep the job. One simply cannot get through life on cognitive ability alone. Even in academia, which is often thought of as among the more cognitive of occupations, one needs to get, and succeed in, job interviews; teach students who do not always want to be taught and teach them in a way that encourages creativity [66]; deal with complaints from students, colleagues, and supervisors; and generally, be able to work oneself through the social networks that determine who is recognized for their work and who is ignored. One scarcely needs formal data to know that some of the most cognitively brilliant people have trouble adjusting to their work environments [9,67].

If one considers a limiting case of an individual with very high cognitive skills but very poor social skills, one could say the individual is “gifted” but has poor social skills. But that individual’s opportunities in life are going to be quite limited. A better way of thinking of them might be to say that they are cognitively gifted but socially limited, and that overall, someone with lesser cognitive skills but more social skills might have more opportunities to succeed in life. The term “gifted” should not be limited to the cognitive domain.

### 2.2. Practical Intelligence

Practical intelligence is the basis for one’s adaptation to, shaping, and selection of environments [9]. Adaptation refers to changing oneself to fit into an environment; shaping refers to changing the environment better to fit oneself; and selection refers to one’s choosing an environment that is suitable for oneself. Selection also has been called niche-finding or niche-picking [68,69]. One finds an environment that is suitable for one’s skills, talents, and preferences.

Practical intelligence depends greatly on tacit knowledge, or what one knows that one does not learn formally and that often is not even explicitly expressed [9,67]. Tacit knowledge generally increases with experience, but what matters most is not so much the amount of experience one has but rather what, and how much, one learns from that experience. Tacit knowledge predicts success in a variety of occupations, such as business manager, salesperson, military officer, and even professor [9,70,71]. Employees at higher levels in an organization tend to have higher tacit knowledge as a result of what they have learned from their experience on the job. Grigorenko and Sternberg [72] found that practical intelligence was a better predictor of physical and mental health than academic intelligence.

Practical-intelligence tests constructed by the Sternberg group are based largely on situational-judgment tests [9]. They generally showed trivial to moderate correlations with measures of general intelligence. In a study in rural Kenya, they showed negative correlations with measures of general intelligence [73]. In that case, students who were viewed as “adaptive” by community standards tended to leave school early in order to work, whereas students who could not get work stayed in school. (This situation may sometimes be true in the post-industrial West as well). The pattern of results shows the extent to which environmental forces can affect correlations between tests of general intelligence and other measures of abilities and accomplishments.

The point regarding correlations of measures with other measures is important to the general point. In the psychometric field and beyond, correlations are often presented as facts of nature: Here is what nature dictates—or some such. If, in contrast, correlations reflect in large part the way societies are constructed, then the correlations in the literature need to be interpreted not as fixed or immutable or somehow “natural”, but rather as products of the interactions of persons with tasks and situations [31].

In general, tests of cognitive abilities will show higher correlations with criteria that require more cognitive load [25]. But suppose that cognitive load is not all or even almost all that is important. For example, the ability to succeed in society may well be correlated with cognitive abilities, but it also undoubtedly reflects social and practical abilities, as developed in the environment [74]. So, the failure of cognitive measures perfectly or nearly perfectly to predict earnings capacity is not actually a failure, but rather an expression of the interaction of those tasks with the environment. In a society that values cognitive abilities more highly, the correlation may be higher; in a society that values them less, or that purges intellectuals because they are viewed as a threat to a totalitarian or other government, the correlation may go down. In Pol Pot’s Cambodia, anyone who was viewed as an intellectual was at risk for their life. In today’s Russia and China, intellectuals with thoughts of their own as to how the government is doing may end up in prison or dead. Both countries have heavy surveillance systems, much like the Soviet Union once had and North Korea appears to have today, that weed out “free thinkers”. Those thinkers are viewed as a threat to repressive, dictatorial, and kleptocratic governments, and with good reason [75,76]. So, where and when higher cognitive abilities may be a threat to societal success, they may also augur societal punishment or expulsion.

The correlation of anything with anything in the literature reflects in part a societal construction that may not exist in other places or at other times. In the days of the feudal dynasties, nothing worked quite as much as station of birth to predict one’s societal success. And to a large extent, this is still true today: Those born into privilege are much more likely to stay with privilege. Thus, in drawing conclusions from correlations, it pays to be intellectually humble in realizing that the correlations are for a certain time and place, not for all times and all places.

Available data suggest, then, that practical intelligence is not merely general intelligence applied to practical affairs, but something different that is largely acquired through experience [9,70]. Tacit knowledge is not something one is born with; it is something one acquires. People can have high IQs but fail to apply those IQs to practical problems. This was a point made some time back by Halberstam [77], who observed that very bright people in government often substantially underperformed what would have been expected of them on the basis of their general intelligence. Janis [78] made a similar point in introducing the concept of groupthink, through which governmental officials considered among the best and brightest of their generation, such as Robert McNamara, made very poor choices when put into groups. IQ without practical intelligence can be dangerous, in part because those with the high IQ may assume that they have practical intelligence they do not have.

### 2.3. Emotional Intelligence

Emotional intelligence is probably the most well-known of the types of intelligence relevant to socioemotional development [79]. Emotional intelligence has been defined as “…the ability to monitor one’s own and others’ feelings and emotions, to discriminate among them and to use this information to guide one’s thinking and actions” [80] (p. 189).

Mayer et al. [59] have pointed out that there are two somewhat distinct conceptions of emotional intelligence. One is based on the original Salovey and Mayer conception and views emotional intelligence as an ability, much as cognitive intelligence is an ability. This conception produces tests that are so-called “maximum performance” tests. One is given a problem to solve that has a right answer and wrong answers, or at the very least, one answer that is substantially better than other answers. The other conception, popularized by Goleman [64,65,74], is a mixed model that views emotional intelligence as more like a personality trait, containing perhaps some ability elements, but more of a matter of an attitude toward life and work. This is the model that, primarily, has given rise to a body of literature on so-called “EQ”. Emotional intelligence, on this model, is measured by typical performance tasks that ask test-takers about themselves, such as how they believe they handle emotionally arousing situations. There are no right-or-wrong answers. If there is a “right” answer, it is the one that best represents how the test-taker truly handles such situations.

The ability-based models predict various external criteria of success generally better than the mixed model but also correlate more highly with conventional ability tests [79]. It is tempting to view the two models as competing, and indeed, this seems to have been the tendency in the literature. But if one believes that most constructs, such as intelligence, critical thinking, creativity, and wisdom, have both ability and attitudinal elements [44,81,82], then the two views are largely complementary rather than contradictory.

## 3. Cognitive Gifts, on the One Hand, and Socioemotional Ones, Are Not Truly Separable

The second assumption of traditional models of giftedness is that the social and emotional are separable from the cognitive. Following up on the above comments on emotional intelligence, one could argue that all the constructs that are used in gifted identification—such as intelligence, and to some extent creativity and wisdom—involve both a cognitive ability and an attitudinal/mindset component. Although one can measure the ability and attitudes separately as an intellectual exercise, in the real world, they are extremely hard to disentangle. For example, many people have critical-thinking abilities, but as soon as new information counters their ideology, religion, or emotional preferences, they fail to use those abilities and may instead act in ways that counter them. Many people who died of COVID-19 did not have to die and would not have, had they not had an ideology that led to their not taking health precautions. When one sees smart people act in stupid ways, one realizes that all the IQ points in the world will not stop people from acting like fools.

Consider a real-world situation in which two hypothetical individuals become pilots. Both have gone through the same training with the same levels of performance, and both have done equally well in simulators. Now, both are in a real-world threat due to weather that challenges their ability to steer the plane to safety. One succeeds; the other does not. The failed pilot was able to handle the simulated threat in the simulator, but not the real-life threat in the real-life situational context. Although this is an extreme case, some of us have experienced something similar with job interviews or auditions. It is one thing to experience the stress of a simulated interview or audition with one’s own group as an audience, but another to experience the actual stress of a real job interview or audition.

One could say that both pilots, or both candidates, had the same abilities but not the same tolerance for the stress of a real-life situation. But the stress of the real-life situation *is* the job. Pilots are hired to fly planes in the real world, not in simulators; musicians are hired to play for real-life audiences, not merely for friends; job applicants of any kind have to face the stress of the actual job, not a simulated job. If one cannot handle “the real thing”, one does not have what it takes for the job.

## 4. Giftedness Is Not Merely inside the Person

The third assumption is that giftedness is, somehow, inside the person. Whatever cognitive or even social or emotional skills are involved, they are seen as properties of the person. So, they are measured as properties of the person by tests of cognitive, emotional, social, practical, or other kinds of intelligence. But, as discussed earlier, all of giftedness, at any level, is an interaction between the person, the task, and the situation [32,83,84,85]. Giftedness is not in the person. A person who is gifted in one kind of task may be totally inept at another kind of task, and how gifted the person is, or whether they are gifted at all, will depend not only on the task, but also on the kind of situation in which the task is confronted. For example, some people work best only if they are at a fairly low level of anxiety, whereas others can get little done unless they feel the pressure of high anxiety in the situation they are dealing with. They are the ones, for example, who only can do the college assignment the night before, when the pressure is at its peak. Thus, when one refers to social and emotional development of the gifted, or even cognitive development of the gifted, there is no meaningful development that can be understood or assessed or even developed outside of task and situational contexts. The idea that any kind of development occurs merely “in the person” does not do justice to the effects of tasks and situations. As noted earlier, the pilot who cannot guide a plane through extremely bad weather might have done fine in good weather. How gifted they are in context depends on the interaction of the pilot with the task of navigating the plane and the situational context of the weather. The same applies to us all. We can steer our way well through some kinds of life circumstances, but probably not others.

In sum, I would frame the problem of “social and emotional development of the gifted” in challenging times, or any times, differently from the way it is usually framed. That development may be part of the giftedness, rather than an adjunct to it; it is not clearly separable from cognitive development; and it is not even clearly separable from the tasks and situations the person will confront.

The issues raised here have been raised in various ways in various contributions to the literature on gifted education. One cannot have a clear conception of how cognitive and social aspects of giftedness differ without a clear conception of what one means by “giftedness”. For example, Siegle et al. [86] have discussed the lack of clarity in definitions of giftedness and gifted identification. One also needs to take into account how one’s upbringing affects cognitive and social development of the gifted. For example, Johnson et al. [87] have discussed parental perspectives on the integration of cognitive competencies into a conception of such competencies fitting into a sociocultural context. Further, one needs to see how the parts fit into a greater whole. For example, Peters et al. [88] have noted the importance of a holistic approach when dealing with students of color and minoritized students. Sociocultural context also affects how cognitive and social skills develop. Alodat et al. [89] have especially pointed out the need, with refugee students, to take into account their divergent backgrounds and skills that may be obscured by common cultural assumptions of the host culture. The fact that the ideas presented here fit into a Zeitgeist of scholars recognizing the need for a more integrated, holistic approach hopefully will lead to some changes in educational practice for those who have taken a questionable approach that involves more “splitting” of gifts and talents from personality and sociocultural context.

Rinn [90], like Siegle et al. [86], has suggested that it is especially important to define and operationalize one’s terms, because one cannot distinguish constructs that are not clearly defined. In discussing psychosocial skills, one needs to ask for whom they are important, when, and under what conditions? Further, psychosocial skills may not be the same when looked at under diverse conditions in the environment. Silverman [91] has noted that development can be asynchronous, whereby individuals with advanced skills have inner experiences and levels of awareness that differ from those of the normal individual. This asynchrony can render the gifted individuals vulnerable, as others may not understand how these individuals perceive the world. The gifted thus need special parental and teacher understanding so that they will not feel, and be seen as, isolated from and perhaps even somewhat alien to others.

Betts and Neihart [92] have pointed out that gifted individuals, however they perceive the world, are not of a unform type. Rather, they argue, gifted individuals can be classified into six different types, who may have different profiles of cognitive and socioemotional skills. For example, what they call “successful” gifted individuals are likely to have well-integrated cognitive and socioemotional skills upon which they can draw to adapt to the environment, whereas “challenging” gifted individuals and “dropouts” may never have reached, in their socioemotional development, a level that well integrates with their cognitive skills. Dai and Chen [93], like others, have pointed out that there are different paradigms for studying giftedness, and that to understand the gifted and how the skills of the gifted operate and integrate, including cognitive and socioemotional skills, one must answer questions of what one means by giftedness, how the gifted operate, who they are, and why they are doing what they are doing.

## 5. Conclusions

In conclusion, first, socioemotional development of the gifted is not separate from giftedness. It is part of giftedness. Second, socioemotional development is not even cleanly and clearly separable from cognitive development. Rather, socioemotional development needs to be viewed as integrated with cognitive development. Third, giftedness and even intelligence, creativity, and wisdom, among other constructs, do not reside inside the person—they are not personal properties but rather interactions of persons with tasks and situations. In sum, giftedness needs to be understood, identified, and taught in a holistic context that encompasses an integration of cognitive, socioemotional, task, and situational contextual elements.

People are not separate, modular systems of cognitive, motivational, affective, and sociocultural processes. Rather, all these processes interact and produce behavior that is not easily predictable as some kind of linear sum of the parts [83]. This article takes the position that, in the field of giftedness, we must treat people as they are, not as divisible parts that can be viewed in isolation from each other.

Why does all this matter? It matters because the problems facing the world have not been well addressed, and certainly not well solved, by people with high IQs who have gone to prestigious colleges and universities. We need to move beyond the idea that any test or set of grades will tell us who will be gifted in what matters—making the world a better place. Giftedness is not well-identified as a matter of a test score and a grade point average. It involves all of the whole person, who will need to navigate their way through the world’s challenges.

## Data Availability

As no data were collected for this work, there are no data to be provided.
